# Brucellar spondylitis occurring in the cervical vertebra: a rare case report and literature review

**DOI:** 10.3389/fmed.2026.1890270

**Published:** 2026-07-21

**Authors:** Junxian Zhang, Jianhui Li, Jun Chen, Yuyao Hao, Yanchao Liu, Shoujun Xie, Hainan Wen

**Affiliations:** 1 Department of Clinical Laboratory, The Affiliated Hospital of Chengde Medical University, Chengde, China; 2 Department of Preventive Medicine, Chengde Medical University, Chengde, China; 3 Department of Geriatrics, The Affiliated Hospital of Chengde Medical University, Chengde, China; 4 Hebei Key Laboratory of Panvascular Diseases, Chengde, China

**Keywords:** *Brucella*, cervical vertebrae, infection, neurological involvement, spondylitis

## Abstract

**Introduction:**

Brucellosis is a zoonotic disease, which can cause systemic infections involving multiple organs. In musculoskeletal diseases, spondylitis is the most common complication of brucellosis, mainly affecting the lumbar and thoracic vertebrae, and rarely involving the cervical vertebrae. Brucellosis-induced cervical spondyloarthritis represents a severe and rare clinical manifestation of brucellosis, potentially leading to permanent damage and neurological dysfunction.

**Case presentation:**

This case report involves a 66-years-old male with a rare case of cervical spondylitis caused by *Brucella*. The diagnosis was based on the patient’s epidemiological history, laboratory tests, and imaging examinations. After triple therapy with doxycycline, rifampicin, and ceftriaxone for the treatment of brucellosis, the patient’s symptoms improved.

**Conclusion:**

Early diagnosis and treatment of spondylitis caused by *Brucella* are very important for the prognosis of patients.

## Introduction

Brucellosis ranks as the most prevalent bacterial zoonosis, with over 500,000 cases around the world each year (1). Brucellosis has exerted a significant negative influence on people’s production and life and has also presented a substantial threat to public health safety. In China, brucellosis is one of the major public health concerns, and the number of human brucellosis cases has increased dramatically (2). *Brucella* mainly infects the human body through contact with sick livestock or contaminated animal products, via the skin, digestive tract, respiratory tract or conjunctiva. Brucellosis has a broad range of clinical presentations, and may manifest as non-specific symptoms such as fever, excessive sweating, joint pain, and general fatigue and pain. In addition to the above symptoms, brucellosis also includes liver damage, bone and joint damage, endocarditis, miscarriage, central nervous system damage, etc. Bone and joint damage represent the predominant and typical complication of brucellosis, affecting 10%–85% of individuals with the infection. The spine is involved in 2%–54% of brucellosis cases, and the incidence rate of cervical vertebrae being affected accounts for 12% of brucellosis-induced spinal inflammation (3, 4). Spondylitis and spondylodiscitis caused by *Brucella* infection primarily affect the lumbar and thoracic vertebrae, while cervical spine involvement is very rare (5). Here we report a case of *Brucella* spondylitis occurring in the cervical spine in the hope of attracting the attention of clinicians and relevant staff to control the spread of brucellosis.

## Case presentation

A 66-years-old male farmer presented with neck and shoulder pain of unclear origin 3 weeks prior. He sought treatment at a local hospital, where he was diagnosed with cervical spondylosis. The patient underwent acupuncture, injection therapy, and rehabilitation. However, 1 day following these interventions, he experienced lower limb weakness and was unable to move. On June 24, 2025, he arrived at the emergency department of Chengde Medical University Affiliated Hospital.

**FIGURE 1:**
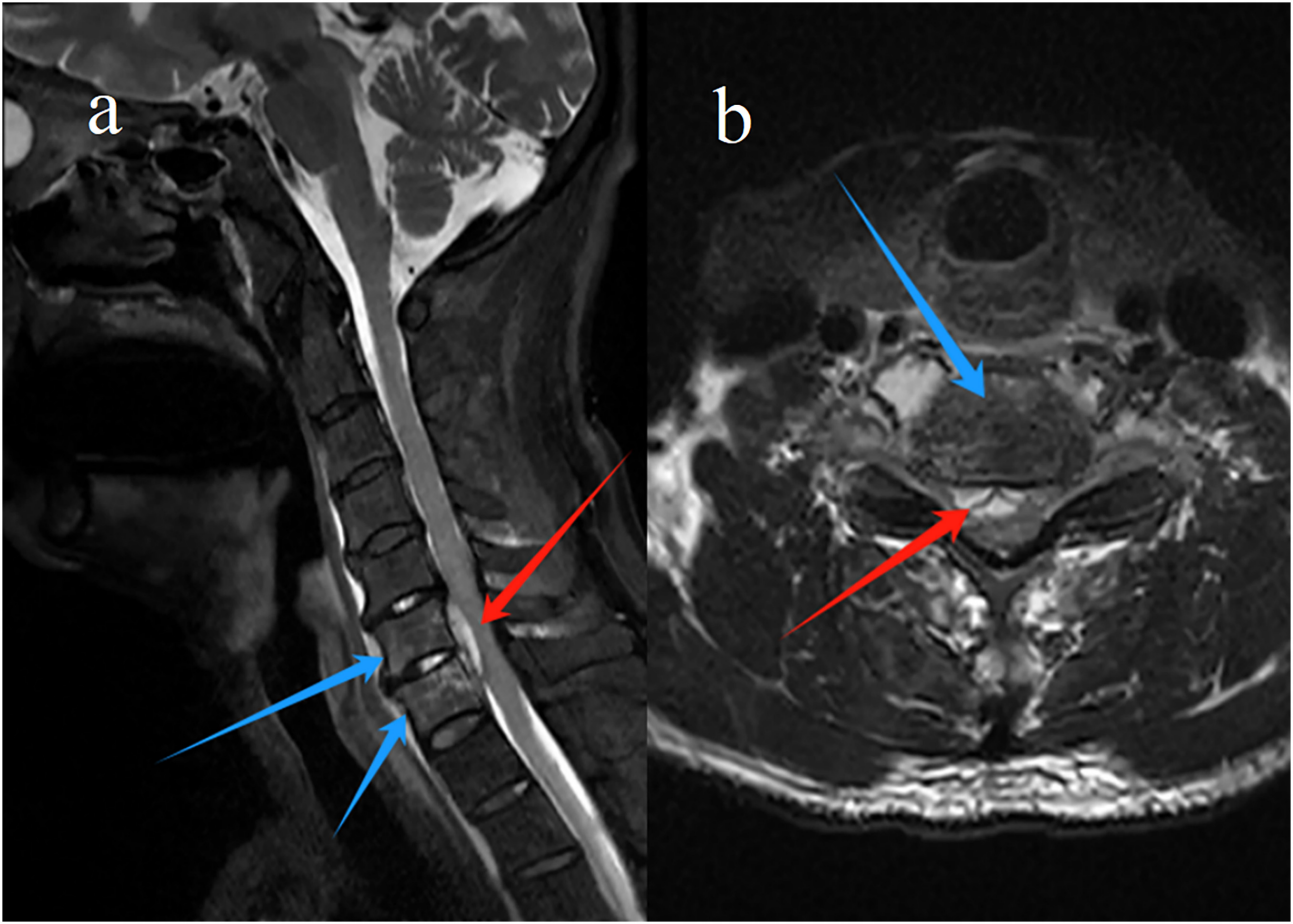
The patient’s magnetic resonance imaging (MRI) examination results. **(a)** The sagittal section scan image of the cervical spine on MRI. The red arrow indicates an epidural abscess, the blue arrow indicates infection of the C6 and C7 vertebrae, and bone marrow edema. **(b)** Axial scan image of medical MRI. The red arrow indicates an epidural abscess, the blue arrow indicates infection of the C6 and C7 vertebrae, and bone marrow edema.

The patient had no prior medical history of chronic diseases such as heart, brain or kidney disorders. Upon admission, the patient’s body temperature was recorded at 36.8°C, with a pulse of 80 beats per minute and a respiratory rate of 18 breaths per minute. Blood pressure measurements indicated 132/67 mmHg. A physical examination revealed tenderness and percussion pain in the interspinous spaces and paravertebral tissues of the cervical and thoracic regions. There was a notable reduction in needle prick pain sensation in the skin of both upper limbs, as well as below three fingerbreadths of both nipples, affecting the trunk and limbs. Additionally, there was diminished needle prick pain sensation in the saddle area and decreased muscle tone in the limbs. Reflexes of the biceps brachii, radial meniscus, and triceps brachii on both sides were weakened, while the knee and Achilles tendon reflexes were absent. The Hoffmann and Babinski signs were negative. Imaging examinations included MRI and CT. The CT results revealed patchy and mixed high-density shadows within the cervical spinal canal at the C5-7 vertebrae. The MRI findings indicated inflammation of the C5/6 vertebral endplate and abnormal signals at the posterior edge of the C6/7 vertebrae, which were associated with spinal canal stenosis. Additionally, hyperplastic changes were observed at the edges of the C3-7 vertebrae and the intervertebral facet joints (Figure 1). Upon admission, the patient’s C-reactive protein (CRP) level was 59.87 mg/L, the white blood cell count measured 9.76 × 10^9^/L, the red blood cell count was 3.06 × 10^12^/L, and the hemoglobin concentration was 90 g/L.

**FIGURE 2:**
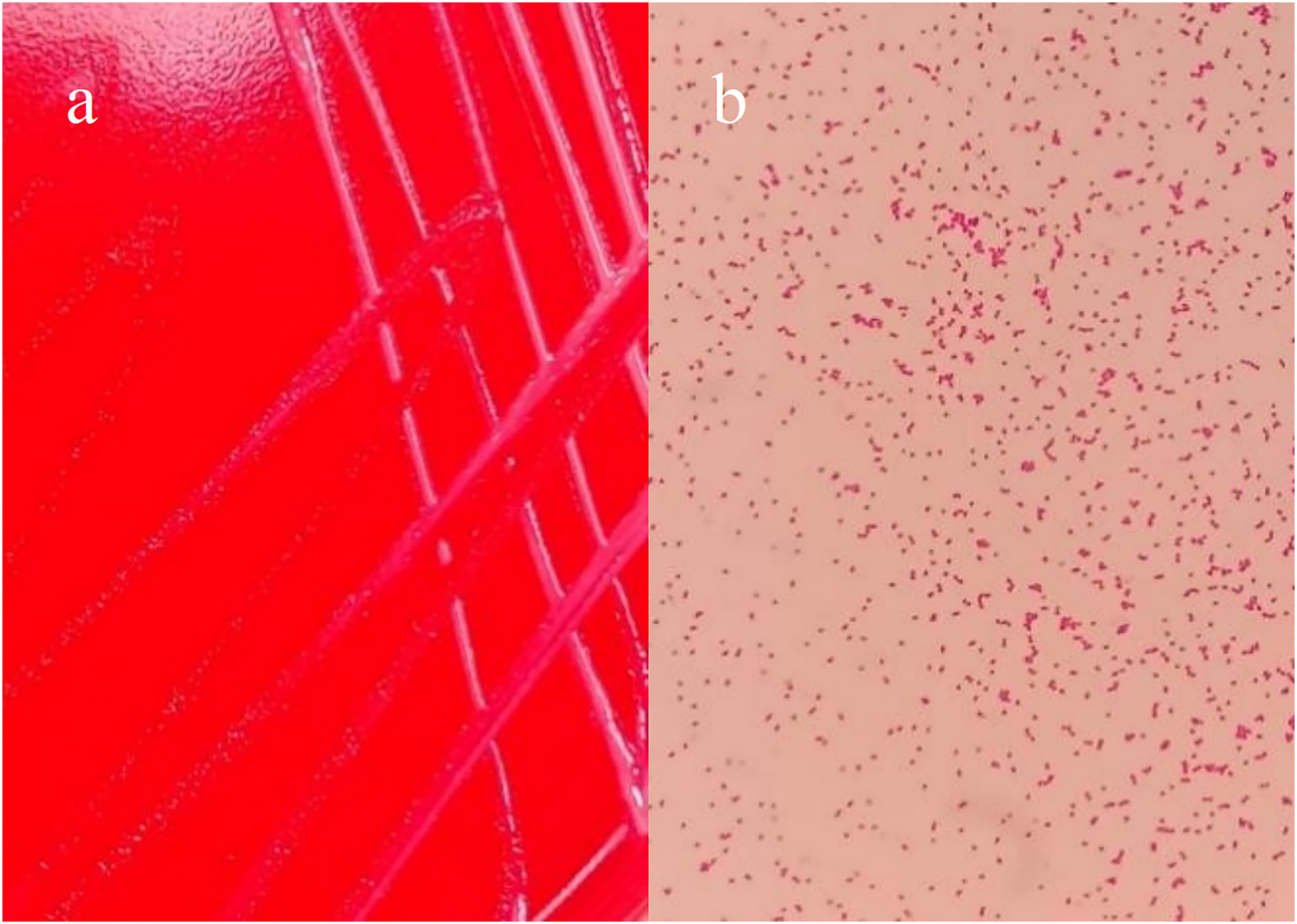
Representative results of Gram staining and sample culture of bacteria. **(a)** Direct smear showed Gram-negative cocci, scattered and in a fine sand-like form, with occasional small clusters. **(b)** After 24 h of cultivation on blood agar medium, the colonies were colorless, translucent, round, smooth in surface, with regular edges, slightly convex in center, and showed no hemolysis.

**FIGURE 3:**
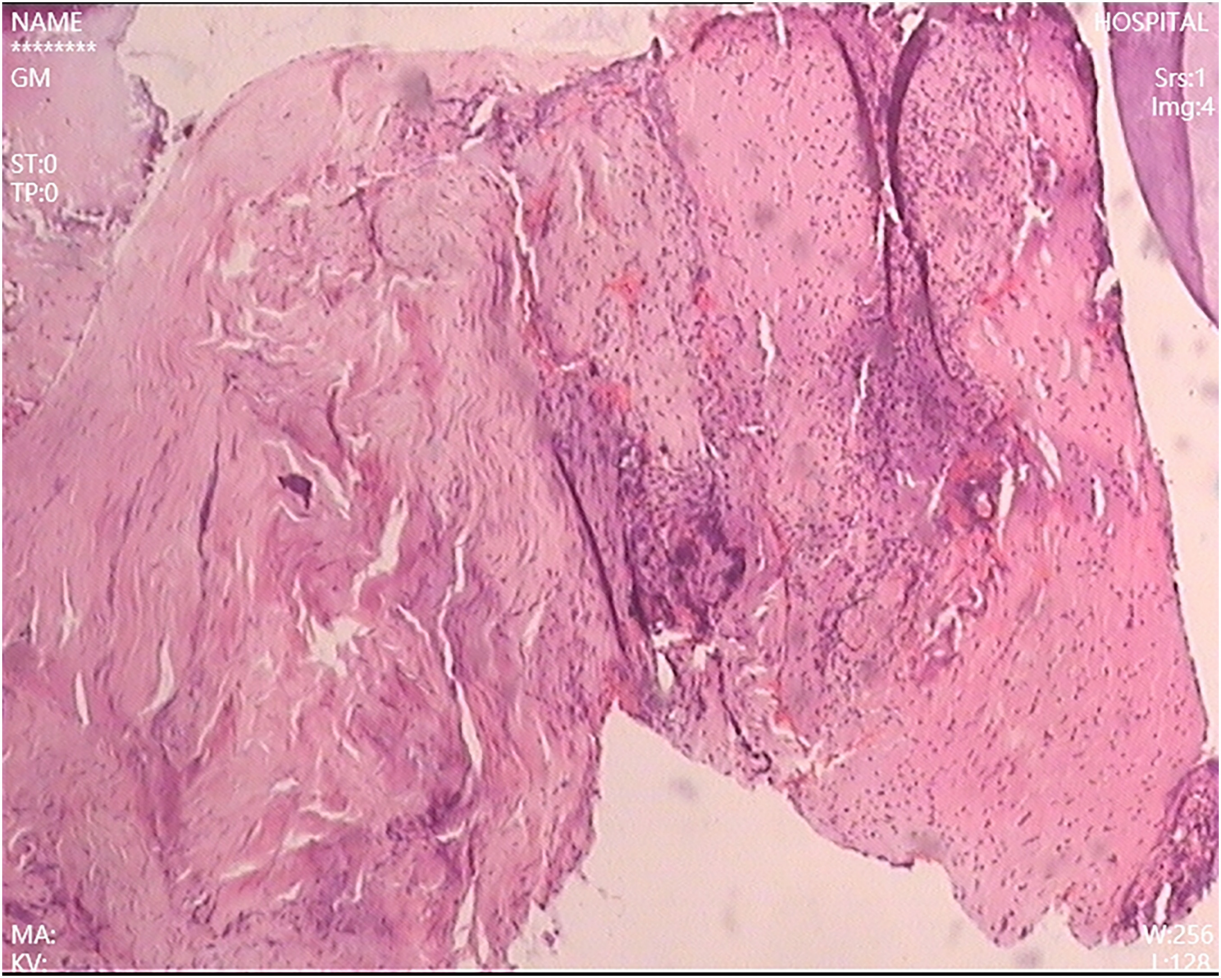
The patient’s pathological results. There was a significant infiltration of neutrophils in the bone marrow tissue.

The patient was admitted to the Spine Surgery Department on June 25, 2025, for posterior cervical lateral mass screw fixation, anterior cervical decompression, abscess removal within the spinal canal, and bone graft fusion with internal fixation. During the procedure, pus was collected for culture. One day after the pus culture, there were small white colonies growing on the blood agar medium. Peripheral blood samples were drawn from both upper limbs on June 25 for blood culture. The blood culture reported positive results after 2.6 days of incubation. The direct smear revealed Gram-negative cocci, which appeared scattered and fine, resembling sand, with occasional clustering into small groups. They are obligate aerobes with high nutritional requirements and slow growth. The colonies are colorless, translucent, round, with a smooth surface, regular edges, a slightly convex center, and no hemolysis (Figure 2). The Matrix assisted laser desorption/ionization time-of-flight mass spectrometry (MALDI-TOF MS) results of the pus culture and blood culture after the operation both indicated the growth of *Brucella*. The pathological examination revealed that there was a significant infiltration of neutrophils in the bone marrow tissue, suggesting a case of suppurative inflammation (Figure 3). This patient has a documented history of contact with cattle and sheep after more interrogation. Based on the patient’s physical examination, cervical magnetic resonance imaging, medical history and laboratory tests, the diagnosis was brucellar spondylitis. Given the unique anatomical location of the brucellar spondylitis affecting the cervical vertebrae, clinicians opted for a comprehensive approach by Sanford Guide to Antimicrobial Therapy and pertinent literature (6, 7). Consequently, a triple-antibiotic regimen comprising doxycycline, rifampicin, and ceftriaxone was selected for therapeutic intervention. After 45 days of treatment, the patient’s inflammatory indicators, including CRP, WBC, PCT, and ESR, exhibited a general decline, ultimately returning to normal levels. Additionally, the incision healed appropriately, and the stitches were removed. Due to the patient’s numbness in the limbs, pain and discomfort in both hip joints and the left knee joint, and limited movement of both lower limbs, the patient was transferred to the rehabilitation department of our hospital. After 28 days of hospitalization, the patient’s limb function improved and the condition was stable, and the patient was discharged and scheduled for regular follow-up.

## Review and discussion

In recent years, it has witnessed a gradual increase of patients with brucellosis along with the development of animal husbandry, with an incidence of 2%–53% (8). The diagnosis of this disease is very difficult, the World Health Organization (WHO) has designated this disease as one of the world’s most important “neglected zoonotic diseases” due to the impact it exerts, especially in low-income nations. In-depth research on *Brucella* and its relationship with animal hosts and humans is of vital significance for the prevention and control of *Brucella* infection. *Brucella* organisms are highly concentrated in the uterus of pregnant animals (9). Animals become infected through ingestion of contaminated feed and water or through direct contact with aborted tissue and uterine secretions. Transmission can also occur via inhalation. One of the most important ways for the transmission of brucellosis among animals is that normal animals share the same water source with *Brucella*-positive animals (10).

Veterinarians, slaughterhouse workers, and shepherds are mainly infected by directly touching the meat or tissues of infected animals with bare hands, splashing infectious fluids onto the skin or conjunctiva, and inhaling aerosols containing the pathogen (11). This patient has a documented history of contact with cattle and sheep. We speculate that cattle and sheep might be the source of the patient’s infection. Following direct exposure to livestock harboring the pathogen or contact with contaminated feed, tools, or clothing, and in the absence of thorough handwashing, the patient may have contracted the infection through behaviors such as rubbing the eyes, eating, or inhaling. *Brucella*, a typical intracellular bacterium, exhibits distinct biological behaviors and immune evasion mechanisms (12). *Brucella* employs virulence factors, including BtpA, BtpB, and Omp25, to disrupt pro-inflammatory signaling pathways and evade the host’s innate immunity (13). This ability allows *Brucella* to survive within macrophages and establish a persistent infection. Ultimately, the bacteria spread through the bloodstream to vascular-rich tissues, such as the spine, thereby laying the pathological groundwork for distal organ lesions, including brucellar spondylitis. Brucellosis of the cervical spine is relatively uncommon, with infection rates differing across various regions. Guenifi et al. conducted a study on patients with *Brucella* vertebral intervertebral discitis in Sétif, Algeria, from January 2016 to December 2022. Their findings indicated that cervical spine brucellosis represented 12% of all spinal brucellosis cases (3). Table 1 summarizes 30 cases of brucellar spondylitis that occurred in the cervical spine and were published since 1982. By analyzing the infection routes of 21 cases (5, 14–33) (excluding those with incomplete information) of brucellosis affecting the cervical spine, it was found that 16 cases were transmitted through the digestive tract. Among them, 15 patients were infected by consuming unpasteurized dairy products, and 1 case was infected by consuming diseased animals. 4 patients were infected through contact, with the main subjects being herdsmen and veterinarians. 1 patient lived in an epidemic area.

**TABLE 1:** Summary of data in 30 cases of cervical *Brucella* spondylitis reported in the literature.

Case	Age/sex	Lesion	Risk factor	Therapeutic drugs and treatment duration	Diagnosis
Zhang et al. (14)	47-year-old male	C5–C6	Consuming unpasteurized dairy products	Doxycycline, rifampicin, levofloxacin and cefotaxime	Serological positivity
Alhusseini et al. (15)	52-year-old male	C6–C7, L3–L4	Consuming milk that has not been pasteurized	Doxycycline, rifampicin and gentamicin for 12 weeks	–
Basaranoglu et al. (30)	50-year-old male	C6–C7	Consuming unpasteurized dairy products	Streptomycin, rifampicin, and doxycycline for 12 weeks	Serological positivity
Hantzidis et al. (49)	65-year-old male	C5–C6	–	Doxycycline and streptomycin for 12 weeks	Serological positivity
Zormpala et al. (16)	38-year-old male	C6–C7, L5–S1	Consuming unpasteurized dairy products	Streptomycin, doxycycline and trimethoprim - sulfamethoxazole for 8 weeks; doxycycline, rifampicin for 2 months	Serological positivity
Resorlu et al. (29)	44-year-old male	C5–C6	Consuming milk that has not been pasteurized	Erythromycin, rifampicin and streptomycin	Both serology and blood culture are positive.
Raptopoulou et al. (31)	71-year-old male	C3–C4, C6–C7, L3–L4, T7–T9	Eating unpasteurized cheese	Streptomycin, doxycycline and trimethoprim-sulfamethoxazole for 8 weeks, dapsone, rifampicin for 3 months	Serological positivity
Pina et al. (41)	75-year-old male	C4–C5	–	Rifampicin, doxycycline and streptomycin for 3 months	Serological positivity
Lampropoulos et al. (5)	70-year-old female	C4–C5	Sheep raising	Streptomycin, rifampicin and doxycycline for 4 months	Serological positivity
Nas et al. (17)	71-year-old male	C3–C4	Consuming unpasteurized dairy products	Rifampicin and doxycycline for 3 months	Serological positivity
Mrabet et al. (18)	56-year-old male	C6–C7, T10–T11, L1–L2	Consuming unpasteurized dairy products	Rifampicin and doxycycline for 3 months	Serological positivity
Roushan et al. (23)	43-year-old male	C6–C7	Living in a popular area	Rifampicin, doxycycline and gentamicin for 3 months	Serological positivity
Wu et al. (19)	27-year-old male	C3–C4	Eating undercooked pork	Doxycycline, rifampicin and levofloxacin	Positive pus culture
Eker et al. (42)	34-year-old male	–	–	Doxycycline and rifampicin for 16 weeks	Both serology and blood culture are positive.
Keenan et al. (20)	45-year-old female	C3–C6, T10–T12, L2–L3	Consuming unpasteurized dairy products	Tetracycline and streptomycin	–
Charalambides et al. (43)	64-year-old male	C3–C4, T9–T10, L2–L3	–	Rifampicin, ciprofloxacin and doxycycline for 12 months	Both serology and blood culture are positive.
Vallalta et al. (21)	41-year-old male	C2–C4	Sheep raising	Doxycycline and ventamycin for 4 months	Serological positivity
Nas et al. (24)	35-year-old female	C1–C2	Consuming unpasteurized dairy products	Doxycycline,trimetoridine/sulfamethoxazole and rifampicin	Serological positivity
Kyebambe et al. (25)	18-year-old girl	C4–C5, C5–C6, C6–C7, C7–T1	Contact with goat feces	Streptomycin, doxycycline and ofloxacin for 20 weeks	Serological positivity
Simsek et al. (44)	6-year-old boy	C1–C5	–	Compound sulfamethoxazole, rifampicin and doxycycline for 6 months	Serological positivity
Sengul et al. (45)	37-year-old male	C7–T1	–	Rifampicin and doxycycline for 6 months	Serological positivity
Dalmak et al. (32)	56-year-old male	C6–C7	Consuming milk that has not been pasteurized	Tetracycline and streptomycin for 3 months	Serological positivity
Tezer et al. (27)	36-year-old female	C3–C4	Consuming unpasteurized dairy products	Streptomycin, doxycycline and trimetoprim-sulfamethoxazole for 6 weeks	Serological positivity
German et al. (22)	36-year-old female	C4–C6	veterinarian	Doxycycline, rifampicin and streptomycin for 3 months	Serological positivity
Emir et al. (28)	6-year-old boy	C5	Consuming unpasteurized dairy products	Trimethoprim-sulfamethoxazole, rifampicin and streptomycin for 6 months	–
Lee et al. (46)	60-year-old male	C5–C6	–	Doxycycline	Both serology and pus culture are positive.
Guzey et al. (47)	61-year-old male	C5–C6	–	Rifampicin, streptomycin and amikacin for 24 weeks	Serological positivity
Basavaraj et al. (26)	60-year-old male	C1–C3, C6–C7	Consumption of unpasteurized goat milk	Doxycycline and streptomycin for 3 months	Serological positivity
Shafizad et al. (48)	36-year-old male	C5–C6	–	Doxycycline and rifampicin for 6 months	Positive pus culture
Tahsin Erman et al. (33)	36-year-old male	C4–C5	Eating unpasteurized cheese	Doxycycline, rifampicin and streptomycin for 6 months	Serological positivity

–, Data not shown.

A study from China has shown that smoking is one of the risk factors contributing to brucellosis (34). The patient has a 50-years history of smoking, averaging 15–20 cigarettes per day, with a cumulative exposure of at least 30 pack-years. This prolonged exposure to tobacco may facilitate *Brucella* infection and the development of spinal arthritis through immunosuppressive mechanisms. Specifically, components such as nicotine in tobacco can impair the phagocytic function of macrophages and diminish the chemotaxis and bactericidal capacity of neutrophils. Consequently, this results in a reduced efficiency of the body’s immune clearance against bacterial pathogens (35). Simultaneously, smoking induces a chronic inflammatory microenvironment within the body. Prolonged smoking can lead to the sustained release of pro-inflammatory cytokines, such as TNF-α and IL-6, while inhibiting the expression of anti-inflammatory mediators like IL-10 (36). This imbalance fosters a pathological and physiological environment that facilitates the immune evasion and persistent infection of *Brucella*. Furthermore, IL-10 plays a crucial role in regulating macrophages, which supports the persistence of *Brucella* and the establishment of chronic infection, as demonstrated in studies utilizing IL-10-deficient mice (37). Additionally, smoking may contribute to vascular damage and bacterial colonization. The vascular endothelial dysfunction associated with smoking may elevate the risk of bacterial adhesion and vascular dissemination (38). The spine, particularly the vertebral endplate area, is more susceptible to bacterial colonization and secondary infection due to its extensive vascular anastomosis network (39).

The diagnostic basis for brucellar spondylitis is the clinical symptoms and signs consistent with the disease (fever, sweating, back pain, hepatosplenomegaly) and a significant titer of specific antibodies (*Brucella* standard tube agglutination [STA] test ≥ 1/100) and/or the isolation of *Brucella* species from blood or biopsy specimens (40). The diagnosis of spinal arthritis caused by *Brucella* is very difficult. The clinical diagnosis analysis of 27 patients (5, 14, 16–19, 21–27, 29–33, 41–49) with brucellar spondylitis revealed that only 6 cases (22.22%) showed positive results in the culture test. Among them, 3 cases were tested with blood culture and 3 cases with pus culture. The detection rate of *Brucella* is relatively low, and there are many difficulties in its culture and testing: this bacterium grows slowly, requires a high-level biosafety laboratory, and has poor sensitivity to chronic or localized infection samples. However, the isolation of this bacterium is indisputable evidence for diagnosing the disease (50). In this case, both the pus culture and blood culture of the patient showed positive for *Brucella*, and the antibody titer of the *Brucella* agglutination test was ≥1:400. Meanwhile, the patient presented with clinical symptoms such as shoulder and neck pain and weakness. Therefore, the patient can be diagnosed with *Brucella* spondylitis. It is worth noting that in this case, the onset site of the patient with *Brucella* spondylitis was in the cervical spine, which is very rare and requires the attention of clinicians. *Brucella* infection damages cervical spine tissues, leading to instability. Furthermore, the associated inflammatory granulation tissue and abscesses can compress the spinal cord. This compression may cause sensory and motor dysfunction in the extremities, potentially resulting in paraplegia with potentially severe consequences.

## Search strategy and selection criteria

A systematic literature search was conducted in PUBMED, EBSCO, SpringerLink, Embase, Web of Science, Cochrane Library. The search terms were (“Brucellosis”[Mesh] OR “*Brucella*”[Mesh]) AND (“Spinal Diseases”[Mesh] OR “Spondylitis”[Mesh] OR “Osteomyelitis”[Mesh]) AND (“Cervical Vertebrae”[Mesh] OR “Neck”[Mesh]), with “spondylitis,” “brucellosis,” “*Brucella*,” “cervical vertebra” as the main keywords. Case reports up to the date of October 20, 2025 were selected, without language or publication year restrictions. The study subjects were patients of any age diagnosed with brucellosis of the spine involving the cervical vertebrae. The only exclusion criterion was inability to obtain the full text.

## Conclusion

Brucellosis-induced spinal arthritis in the cervical vertebrae represents a severe and rare clinical manifestation of brucellosis, potentially resulting in permanent damage and neurological dysfunction. The timely diagnosis of brucellar spondylitis remains a clinical challenge. In this case, we detected *Brucella* in the pus and blood samples promptly, providing an accurate etiological basis for the subsequent effective treatment in clinical practice. Therefore, it is of great significance to accurately identify this bacterium at an early stage. Microbiological laboratories need to continuously improve their detection capabilities for such pathogenic bacteria.

## Data Availability

The original contributions presented in this study are included in the article/supplementary material, further inquiries can be directed to the corresponding author.
